# In-hospital predictors of post-stroke depression for targeted initiation of Selective Serotonin Reuptake Inhibitors (SSRIs)

**DOI:** 10.1186/s12888-022-04378-0

**Published:** 2022-11-19

**Authors:** Julie Yi, Justin Lu, Annie Yang, Elisabeth Breese Marsh

**Affiliations:** Department of Neurology, Johns Hopkins University School of Medicine, 600 North Wolfe St. Phipps 446C, Baltimore, MD 21287 USA

**Keywords:** Stroke, Depression, Outcomes, Recovery

## Abstract

**Background:**

Although SSRIs are no longer widely prescribed for post-stroke motor recovery, fluoxetine demonstrated beneficial effects on post-stroke depression (PSD). Given the potential side effects of SSRIs, targeted initiation among individuals at highest risk for PSD warrants consideration. While previous studies have identified stroke severity and psychiatric history as factors associated with PSD, its predictability remains unknown. In this study, we investigate inpatient predictive factors to better identify individuals who might derive the most benefit from targeted initiation of SSRIs.

**Methods:**

We performed a retrospective analysis of a prospectively-collected registry of adult patients presenting with acute ischemic stroke to a tertiary referral urban academic comprehensive stroke center between 2016–2020. Patients were seen 4–6 weeks post-discharge and administered the PHQ-9 (Patient Health Questionnaire-9) to screen for PSD (PHQ-9 ≥ 5). Demographics, history of depression, stroke severity, and inpatient PHQ-9 scores were abstracted. Logistic regression was used to determine factors associated with PSD and an ROC analysis determined the predictability of PSD in the inpatient setting.

**Results:**

Three hundred seven individuals were administered the PHQ-9 at follow-up (mean age 65.5 years, 52% female). History of depression (OR = 4.11, 95% CI: 1.65–10.26) and inpatient PHQ-9 score (OR = 1.17, 95% CI: 1.06–1.30) were significantly associated with PSD. Stroke severity, marital status, living alone, employment, and outpatient therapy were not associated with PSD. The ROC curve using a positive inpatient PHQ-9 achieved an area under the curve (AUC) of 0.65 (95% CI: 0.60–0.70), while the AUC was 0.72 (95% CI: 0.66–0.77) after adding history of depression.

**Conclusions:**

History of depression and a positive inpatient PHQ-9 appear to be most strongly predictive of long-term PSD. Initiating SSRIs only in those individuals at highest risk for PSD may help reduce the burden of stroke recovery in this targeted population while minimizing adverse side effects.

## Background

Stroke affects nearly 800,000 people in the US each year, and is a leading cause of long-term disability [1]. Neuropsychiatric disorders after stroke are common and debilitating, including post-stroke depression (PSD) [2–4]. Despite its prevalence ranging from 29–39% with a cumulative incidence of 55% [5], PSD is often underdiagnosed and poorly treated [4, 6]. Given that PSD is associated with worse functional outcomes and higher mortality after stroke [6, 7], early identification and aggressive treatment of post-stroke depressive symptoms may help reduce the overall burden of stroke recovery.

Among pharmacologic agents used in stroke recovery, fluoxetine, a selective serotonin reuptake inhibitor (SSRI), was initially thought to promote motor recovery after stroke by enhancing neuroplasticity and promoting neurogenesis [8, 9]. However, subsequent clinical trials (FOCUS [﻿Fluoxetine or Control Under Supervision) [10], AFFINITY [﻿Assessment of Fluoxetine in Stroke Recovery] [11], EFFECTS [﻿Efficacy of Fluoxetine—a Randomized Controlled Trial in Stroke] [12]) suggested limited clinical utility of fluoxetine on functional outcomes after stroke. Although the benefit of SSRIs on functional outcomes after stroke is uncertain, fluoxetine demonstrated a beneficial effect on mood and emotional control in both FOCUS and EFFECTS, with lower rates of new onset depression in patients receiving fluoxetine [10, 12].

Given that SSRIs are not without potential side effects including bone fractures [12] and falls [11], selective initiation among individuals at highest risk for PSD warrants consideration. While previous studies have identified stroke severity and psychiatric history as factors associated with PSD [13–15], the predictability of PSD in the inpatient setting remains unknown. Identifying inpatient predictors of PSD may inform future stroke recovery interventions and help target a population who may benefit from early intervention. The primary purpose of this study was to investigate inpatient predictive factors for PSD to better identify individuals who might derive the most benefit from early initiation of SSRIs. As a secondary objective, we examined the change in PHQ-9 (Patient Health Questionnaire 9) score at hospitalization and 4–6 weeks post-discharge among those prescribed SSRIs in the post-stroke recovery period to understand the impact of SSRIs on PSD.

## Methods

### Patient selection

Adult patients presenting with acute ischemic stroke to a tertiary referral urban academic comprehensive stroke center between October 2016 to December 2020 were enrolled in a prospectively-collected Institutional Review Board (IRB)—approved stroke registry. Following approval from the Johns Hopkins School of Medicine IRB, this study retrospectively analyzed a subset of registry patients (18 years and older) hospitalized for acute ischemic stroke who were seen in outpatient follow-up clinic 4–6 weeks post-discharge and administered the PHQ-9 to evaluate for post-stroke depression. Given the observational nature of the study, informed consent was not required. Patients with acute hemorrhagic stroke and those without a post-discharge PHQ-9 score at 4–6 weeks were excluded. Patients who returned for follow-up 4–6 weeks post-discharge were compared to those lost to follow-up to identify any potential selection bias.

### Data collection

Demographic variables, marital status, living arrangements, and employment status were identified through electronic medical records within the Department of Neurology. History of stroke, history of depression, prior use of an SSRI, stroke severity using the National Institutes of Health Stroke Scale (NIHSS) at the time of stroke diagnosis, and inpatient PHQ-9 score were identified from the most recent ischemic stroke hospitalization for each patient. Post-stroke SSRI use, patient-reported post-stroke depressive symptoms, and participation in outpatient rehabilitation therapy were abstracted from follow-up clinic documentation. Post-discharge PHQ-9 score at 4–6 weeks was identified through the stroke registry. For this study, the following medications were included as SSRIs (or similar SNRI): citalopram, duloxetine, escitalopram, fluoxetine, nortriptyline, paroxetine, sertraline, venlafaxine, and vortioxetine.

### Statistical analysis

Differences between individuals with and without PSD were retrospectively compared using the Wilcoxson Rank Sum test for continuous variables and ﻿chi-squared test for categorical variables. The primary outcome of interest was PSD at follow-up. This diagnosis was made by a board-certified neurologist with experience in diagnosing and treating PSD, using the PHQ-9 as an initial screen, where scores below 5 indicate minimal depression while scores greater than or equal to 5 suggest mild, moderate, or severe depression [16]. A score of 5 or more prompted automatic clinical evaluation for PSD, though depression is typically discussed at all clinic visits regardless of screening score. No patient with a score of 5 or more at follow-up was not ultimately diagnosed with PSD, and so a cut-off of 5 on the PHQ-9 was used as a proxy for PSD in this study. Unadjusted logistic regression was used to determine factors associated with PSD. We subsequently created a multivariable adjusted model including age, sex, race, marital status, living situation, employment, history of stroke, history of depression, prior SSRI use, NIHSS, inpatient PHQ-9 score, and outpatient therapy, with all listed measures entered simultaneously into the model. Factors demonstrating the strongest association with PSD (*p*-value < 0.05) were included in Receiver Operating Characteristic (ROC) analysis to determine the predictability of PSD in the inpatient setting. A *p* value equal to or less than 0.05 was considered statistically significant. All analyses were performed using Stata 17 (StataCorp LP, College Station, TX).

The secondary outcome of interest was change in PHQ-9 score defined as the difference between the inpatient PHQ-9 score and PHQ-9 score at 4–6 weeks post-discharge, as a continuous variable. To understand the impact of taking SSRIs after discharge on PSD, multivariable-adjusted linear regression was used to estimate the magnitude of the association between post-stroke SSRI and change in PHQ-9 score stratified by inpatient PHQ-9 score (positive score defined as greater than or equal to 5). We adjusted for potential confounders including age, sex, race, marital status, living situation, employment, history of stroke, history of depression, prior SSRI use, NIHSS, and outpatient therapy. We additionally adjusted for whether the individual was taking an SSRI at the time of admission.

## Results

### Patient characteristics

Three hundred seven patients admitted for acute ischemic stroke were administered the PHQ-9 4–6 weeks post-discharge. This cohort was younger (mean age 65.5 vs. 68.6) with less severe strokes (mean NIHSS 5.2 [median 3, IQR 1–7] vs. 8.5 [median 6, IQR 2–14]), but had no difference in history of depression or inpatient PHQ-9 scores than those who did not return to clinic for follow-up. Otherwise, there were no significant differences between groups. The mean age (SD, range) of the cohort was 65.5 (15.2, 19 to 101). 158 (51.5%) were female and 84 (27.4%) self-identified as Black. The mean NIHSS score (SD) was 5.2 (5.7) in the total cohort. One hundred sixty-seven (54.4%) had documented PSD at follow-up. The mean post-stroke PHQ-9 score (SD) was 1.2 (1.3) among those without PSD and 11.3 (5.2) among those with PSD (Table 1).

**Table 1:** Baseline demographic and clinical characteristics stratified by post-stroke depression (PHQ-9 ≥ 5)

	**Total Cohort (*N* = 307)**	**PHQ-9 < 5 (*N* = 140)**	**PHQ-9 ≥ 5 (*N* = 167)**	*P* value *
**Demographics**				
Age, mean (SD) (yr)	65.5 (15.2)	66.5 (15.4)	64.8 (15.0)	0.25
Sex (female), n (%)	158 (51.5)	70 (50.0)	88 (52.7)	0.64
Race				
White, n (%)	210 (68.4)	93 (66.4)	117 (70.1)	0.68
Black, n (%)	84 (27.4)	42 (30.0)	42 (25.2)	
Asian, n (%)	4 (1.3)	1 (0.7)	3 (1.8)	
Native American, n (%)	2 (0.7)	1 (0.7)	1 (0.6)	
Not reported, n (%)	7 (2.3)	3 (2.1)	4 (2.4)	
Married, n (%)	131 (42.7)	63 (45.0)	68 (40.7)	0.45
Lives alone, n (%)	49 (16.0)	17 (12.1)	32 (19.2)	0.10
Employed, n (%)	120 (39.1)	62 (44.3)	58 (34.7)	0.12
**Medical history**				
History of stroke, n (%)	63 (20.5)	30 (21.4)	33 (19.8)	0.72
History of depression, n (%)	85 (27.7)	18 (12.9)	67 (40.1)	< 0.001
Prior SSRI use, n (%)	58 (18.9)	13 (9.3)	45 (27.0)	< 0.001
Admission SSRI use, n (%)	38 (12.4)	5 (3.6)	33 (19.8)	< 0.001
**Stroke Admission**				
NIHSS, mean (SD)^a^	5.2 (5.7)	4.4 (5.1)	5.8 (6.1)	0.04
Inpatient PHQ-9 score, mean (SD)^b^	4.0 (4.7)	2.4 (3.0)	5.5 (5.5)	< 0.001
**Stroke recovery**				
Post-stroke SSRI, n (%)	132 (43.0)	50 (35.7)	82 (49.1)	0.02
Subjective depression, n (%)	63 (20.5)	10 (7.1)	53 (31.7)	< 0.001
Outpatient therapy, n (%)	62 (20.2)	26 (18.6)	36 (21.6)	0.52
1-month post-stroke PHQ-9, mean (SD)	6.7 (6.4)	1.2 (1.3)	11.3 (5.2)	< 0.001

^*^P-values are from chi-square (categorical variables) and Wilcoxson Rank Sum test (continuous variables) for differences across groups

^a^*N* = 293

^b^*N* = 244

### Predictors of post-stroke depression

The proportion of patients with history of depression and prior SSRI use was higher among individuals with PSD (40.1% and 12.9%, respectively) compared to those without PSD (24.0% and 7.9%, respectively). Inpatient PHQ-9 scores were also higher among those with PSD compared to those without PSD (5.5 [5.5] vs. 2.4 [3.0]).

In unadjusted analyses, individuals with a history of depression (OR = 4.54, 95% CI: 2.53–8.14) and prior SSRI use (OR = 3.60, 95% CI: 1.85–7.01) had greater odds of PSD (Table 2). A higher NIHSS score was associated with 1.05 (95% CI: 1.00–1.10) higher odds of PSD. A higher inpatient PHQ-9 score was associated with 1.19 (95% CI: 1.11–1.28) higher odds of PSD. Marital status, living alone, employment, and outpatient therapy were not associated with PSD. In the multivariable-adjusted model, history of depression (OR = 4.11, 95% CI: 1.65–10.26) and inpatient PHQ-9 score (OR = 1.17, 95% CI: 1.06–1.30) remained significantly associated with PSD. The ROC curve using a positive inpatient PHQ-9 achieved an area under the curve (AUC) of 0.65 (95% CI: 0.60–0.70), while the AUC was 0.72 (95% CI: 0.66–0.77) after adding history of depression.

**Table 2:** Estimated associations between demographic/clinical variables and post-stroke depression (PHQ-9 ≥ 5) at follow-up (*N* = 307)

	**OR (95% CI)**	***P* value**	**Adjusted OR (95% CI)**^**a**^	***P* value**
**Demographics**				
Age	0.99 (0.98–1.01)	0.335	0.98 (0.96–1.00)	0.11
Sex (female)	1.11 (0.71–1.75)	0.638	0.72 (0.37–1.43)	0.35
Race				
White	1		1	
Black	0.79 (0.48–1.32)	0.375	0.75 (0.35–1.62)	0.47
Asian	2.38 (0.24–23.30)	0.455	0.56 (0.01–55.12)	0.81
Native American	0.79 (0.05–12.88)	0.872	-	-
Married	0.84 (0.53–1.32)	0.450	0.87 (0.43–1.78)	0.71
Lives alone	0.59 (0.31–1.11)	0.102	0.60 (0.25–1.46)	0.26
Employed	0.69 (0.43–1.11)	0.123	0.58 (0.27–1.23)	0.16
**Medical history**				
History of stroke	0.76 (0.52–1.57)	0.719	0.73 (0.33–1.71)	0.50
History of depression	4.54 (2.53–8.14)	< 0.001	4.11 (1.65–10.26)	0.002
Prior SSRI use	3.60 (1.85–7.01)	< 0.001	1.51 (0.52–4.43)	0.45
**Stroke admission**				
NIHSS	1.05 (1.00–1.10)	0.032	1.05 (0.97–1.14)	0.21
Inpatient PHQ-9 score	1.19 (1.11–1.28)	< 0.001	1.17 (1.06–1.30)	0.002
**Stroke recovery**				
Outpatient therapy	1.20 (0.69–2.12)	0.517	1.54 (0.71–3.36)	0.28

^**a**^Adjusted for age, sex, race, marital status, living situation, employment, history of stroke, history of depression, prior SSRI use, NIHSS, inpatient PHQ-9 score, and outpatient therapy

### Impact of SSRIs on post-stroke depression

The difference in change in PHQ-9 score between individuals with and without post-stroke SSRI was -4.12 (95% CI: -8.04 to -0.19) among individuals with inpatient PHQ-9 score ≥ 5, suggesting that post-stroke SSRI is associated with improvement in depressive symptoms at follow-up among individuals screening positive on the inpatient PHQ-9 (Table 3). Individuals with higher inpatient PHQ-9 scores exhibited greater improvement in PHQ-9 scores at follow-up (Table 3).

**Table 3:** Multivariable-adjusted change in PHQ-9 score by inpatient PHQ-9 score

	**Inpatient PHQ-9 < 5 (*N* = 167)**		**Inpatient PHQ-9 ≥ 5 (*N* = 140)**	
	**Estimate (95% CI)**^a^	***P* value**	**Estimate (95% CI)**^a^	***P* value**
Post-stroke SSRI	0.39 (-1.60 to 2.37)	0.700	-4.12 (-8.04 to -0.19)	0.040

^a^Adjusted for age, sex, race, marital status, living situation, employment, history of stroke, history of depression, prior SSRI use SSRI, admission SSRI use, NIHSS, and outpatient therapy

## Discussion

Among patients with acute ischemic stroke who presented for follow-up 4–6 weeks post-discharge, history of depression and inpatient PHQ-9 scores were identified as significant in-hospital predictors of PSD. These results suggest that individuals with a history of depression and those reporting depressive symptoms during hospitalization deserve additional consideration for targeted treatment with antidepressants. Depressive symptoms in the inpatient setting are not transient, and should be taken seriously as a risk factor for PSD, which can further exacerbate stroke recovery when left untreated [4]. Our study also suggests that for patients with depressive symptoms during hospitalization, initiation of SSRIs in the post-stroke recovery period may have the potential to reduce depressive symptoms at 4–6 weeks post-discharge. In weighing the risks and benefits of SSRIs in stroke recovery, patients with a history of depression reporting depressive symptoms in the inpatient setting may stand to reap the most benefit from targeted initation of SSRIs.

The predictors of PSD identified in our study extend the literature on previous studies reporting risk factors for PSD. Although history of depression and stroke severity have been identified in previous studies as risk factors for PSD [4, 17–19], in-hospital depressive symptoms using the PHQ-9 have not yet been described as a predictor in the literature. Depressive symptoms during hospitalization deserve adequate attention for evaluation and treatment. These symptoms should not be routinely dismissed as a transient reaction to the immediate disabilities precipitated by stroke. In addition, inpatient PHQ-9 score may contribute additional clinical utility since history of depression assessed as a binary variable does not capture the temporality of depressive symptoms leading up to hospitalization for stroke. Including the inpatient PHQ-9 in clinical decision-making may help identify a group of individuals experiencing more severe depressive symptoms that are acutely precipitated by stroke and deserve targeted treatment. Our finding that stroke severity was not significantly associated with post-stroke depression in our multivariable-adjusted model may be explained by the fact that those returning for follow-up had less severe strokes with a smaller range. Although not statistically significant, the trend observed in our study was consistent with previous studies suggesting increased stroke severity as a risk factor for post-stroke depression [4, 17–19]. Factors not associated with PSD in our study were also consistent with previous studies; these included older age, sex, stroke subtype, level of education, living alone, marital status, or a previous stroke [17, 18].

Our study suggests that SSRIs may have the potential to improve depressive symptoms at 4–6 weeks post-discharge, adding to the growing body of literature on antidepressant use in stroke recovery. We found that among patients with higher inpatient PHQ-9 scores, initiation of SSRI in the post-stroke setting was associated with greater improvement in PHQ-9 at 4–6 weeks follow-up compared to no SSRI. This trend was not observed among patients with PHQ-9 scores less than 5. Available evidence suggests that pharmacological interventions including SSRIs may prevent depression and improve mood after stroke, although the certainty of evidence is limited to make definitive recommendations on routine prescription of SSRIs [20]. In a randomized controlled trial investigating prevention of depression among patients within three months of stroke onset, the risk of new-onset depression for patients assigned to placebo was more than four times greater than the risk for patients treated with escitalopram [21]. A meta-analysis of 8 randomized RCTs investigating the efficacy of preventive interventions for PSD found that the likelihood of developing PSD was reduced with the use of an SSRI among individuals without depressive symptoms at baseline. Furthermore, there were no significant differences in the frequency of side effects (e.g., nausea, diarrhea, fatigue, and dizziness) between those on active treatment and placebo [22]. In contrast, recent evidence on depression outcomes among patients treated with fluoxetine in the ﻿AFFINITY trial found that routine daily treatment with 20 mg fluoxetine did not decrease the proportion of people affected by PSD compared to placebo [23]. Our finding that the improvement in depressive symptoms was notable only for individuals with PHQ-9 greater than or equal to 5 may explain the contrasting result from the AFFINITY trial [23]. In the AFFINITY trial, the mean (SD) PHQ-9 score in the fluoxetine and placebo groups were below 5 at 4.8 (4.3) and 4.9 (4.1), respectively. Stratifying by severity of depressive symptoms in the inpatient setting and selectively initiating SSRIs among patients with higher PHQ-9 scores may help reduce the burden of stroke recovery while minimizing risk of adverse side effects.

Given that the current state of the evidence for SSRIs on PSD does not support routine prescription of SSRIs in stroke recovery, our study lends support for a targeted approach of initiating SSRIs where benefit clearly outweighs the potential risk of harm. Despite the fact that PSD is treatable, the prevalence of PSD has not decreased significantly over the past decade [24]. Therefore, targeted strategies based on clinical predictors of PSD presented in this study are needed. Further research to better understand the duration and dosage of SSRIs needed to maximize benefit in a targeted population may ultimately help reduce rates of PSD.

Our study is not without limitations. We included patients who followed up at a single multidisciplinary stroke center 4–6 weeks post-discharge who were able to complete the PHQ-9. Given significant disability after stroke, these patients are often accompanied by their care partners and need assistance with transportation and other logistical coordination to present to the health care setting. Patients who may not have the necessary social support to navigate the health care system may have been disproportionately lost to follow-up. There may have been selection bias towards the null if patients with more severe strokes or depressive symptoms were lost to follow-up. Conversely, patients who followed up in the outpatient setting may have been those who experienced more difficulty with post-stroke recovery and therefore actively sought medical care. Patients with a history of depression or those who were previously treated with SSRIs may have been more engaged with the health care system in the stroke recovery period. Although we recognize the potential for selection bias in this study, analysis comparing patients with PHQ-9 scores 4–6 weeks post-discharge with those who did not follow-up demonstrated no significant differences in terms past medical history of depression or inpatient PHQ-9 scores. In addition, we did not assess SSRI adherence in this study. We used SSRI mentioned in the follow-up documentation medication list as a proxy for taking an SSRI. Low adherence may have underestimated the impact of SSRIs on PHQ-9 scores 4–6 weeks post-discharge. Although we used data from a prospectively-collected stroke registry, we identified predictors of PSD using a retrospective analysis in a single cohort of acute ischemic stroke patients presenting to a tertiary academic health center. Prospective validation in an external dataset is needed to reliably identify predictors of PSD in other acute ischemic stroke recovery populations. In addition, our inclusion criteria required that patients have the appropriate cognitive abilities to complete the PHQ-9. Given that the prevalence of post-stroke cognitive impairment ranges from 7.4% after a first stroke to 41.3% in recurrent stroke [25], patients with more severe post-stroke cognitive impairment may not have reliably completed the PHQ-9. Lastly, our results are not generalizable to patients with hemorrhagic stroke as we only included patients presenting with acute ischemic stroke. Despite these limitations, our study extends the literature on stroke recovery by identifying predictors of PSD that can be used to better identify and target at-risk individuals for initiation of SSRIs.

Given that PSD is highly prevalent, affecting 1 in 3 individuals during the first year after stroke [24], the ability to predict PSD at time of hospitalization would potentially enable targeted initiation of SSRIs to maximize the benefit of improving mood and minimize the harm associated with side effects. History of depression and inpatient PHQ-9 scores are in-hospital predictors of PSD that can be readily assessed in the inpatient setting to aid clinical decision-making. Furthermore, among patients screening positive for depressive symptoms in the inpatient setting, SSRIs may have the potential to improve depressive symptoms at 4–6 weeks follow-up.

## Conclusions

Although the clinical decision to initiate SSRIs in the post-stroke setting depends on the unique circumstances for each patient, initiating SSRIs particularly among those at risk for PSD is an important step in reducing the overall burden of stroke recovery. History of depression and an inpatient PHQ-9 score greater than or equal to 5 appear to be most strongly predictive of PSD at 4–6 weeks post-discharge and should be strongly weighted in treatment decisions. Therefore, targeted initiation of SSRIs only among patients with a history of depression and those exhibiting depressive symptoms in the inpatient setting may maximize the benefit of SSRIs while minimizing the risk of side effects.

## Data Availability

Data will be made available upon reasonable request to the PI.

## Abbreviations

SSRI: Selective serotonin reuptake inhibitor
PSD: Post-stroke depression
PHQ-9: Patient Health Questionnaire-9
AUC: Area under the curve
IRB: Institutional review board
NIHSS: National Institutes of Health Stroke Scale
SNRI: Seratonin and norepinephrine reuptake inhibitor
ROC: Reciever operating characteristic
SD: Standard deviation
